# Genetic, hormonal, and transcriptomic analyses highlight the crucial role of phytohormones in first branch angle regulation in pepper

**DOI:** 10.1186/s12870-025-07499-2

**Published:** 2025-11-03

**Authors:** Xinjie Yuan, Xuejun Chen, Kunhua Zhou, Gang Lei, Yueqin Huang, Gege Li, Yu Fang, Jubin Wang, Rong Fang

**Affiliations:** 1 Institute of Vegetables and Flowers, Jiangxi Academy of Agricultural Sciences, Nanchang, Jiangxi 330200 China; 2 Jiangxi Provincial Key Laboratory of Horticultural Crops (Fruit, Vegetable & Tea) Breeding/Jiangxi Provincial Engineering Research Center for Vegetable Molecular Breeding/Biological Breeding Innovation Center of Jiangxi Province, Jiangxi Academy of Agricultural Sciences, Nanchang, Jiangxi 330200 China; 3 Jiangxi Provincial Key Laboratory of Improved Variety Breeding and Efficient Utilization of Native Tree Species, Jiangxi Academy of Sciences, Nanchang, Jiangxi 330299 China

**Keywords:** First branch angle, Phytohormone, GWAS analysis, Transcriptomic analyses, Pepper (*Capsicum annuum* L.)

## Abstract

**Background:**

The first branch angle (FBA) is a critical trait influencing plant architecture, yield, and mechanized harvesting efficiency in pepper (*Capsicum annuum* L.). However, the genes and regulatory processes involved remain largely unclear.

**Results:**

The phenotypic evaluation of 220 pepper accessions revealed significant genetic variation in FBA, with a broad-sense heritability of 93.26%, indicating a strong genetic foundation and making it a stable trait for selection. A genome-wide association study (GWAS) with mixed linear model and FarmCPU detected 25 significant SNPs related to FBA. Several candidate genes were identified, encompassing components of brassinosteroid signaling, gibberellin signaling, serine/threonine-protein kinase signaling, and cell wall modification. To functionally contextualize these genetic findings, two accessions with divergent FBAs (compact B010 and loose B003) were selected for cytological and hormonal analyses. The paraffin sections stained with Periodic Acid-Schiff showed that the compact B010 exhibits more starch granules in endodermal amyloplasts, while the loose B003 has larger cell size on the adaxial side. Hormonal analysis based on HPLC-MS/MS revealed higher auxin levels in the compact B010 and higher gibberellin, cytokinin, brassinosteroid, and strigolactone levels in the loose B003. These results suggest a mechanism whereby brassinosteroid/gibberellin-mediated cell expansion, potentially through the action of cell wall modification genes, drives the loose architecture of B003. Furthermore, RNA-Seq and qRT-PCR confirmed the coordinated regulation of the key phytohormone pathways (auxin signaling; cytokinin, gibberellin, and brassinosteroid biosynthesis) and serine/threonine-protein kinase signaling. Therefore, our work integrates multi-layered analyses to propose a regulatory framework for FBA in pepper: genetic loci associate with alterations in phytohormone regulation, kinase activity, and cellular traits, which orchestrate the transcriptomic, hormonal, and cytological changes that collectively determine branch architecture.

**Supplementary Information:**

The online version contains supplementary material available at 10.1186/s12870-025-07499-2.

## Introduction

Pepper (*Capsicum* spp.), a globally cultivated crop within the Solanaceae family with increasing economic importance, serves culinary, medicinal, ornamental, and spicing purposes [1, 2]. In 2023, global pepper production reached 38.31 million tons of fresh fruit and 5.82 million tons of dried pods, cultivated over 3.87 million hectares (www.fao.org). Pepper plant architecture is crucial in influencing both overall yield and the efficiency of mechanized harvesting. In the sympodial shoot structure of pepper, the main stem stops elongating after reaching a certain height. At this stage, the shoot apical meristem transitions into an inflorescence meristem, shifting growth initiation to lateral meristems, known as sympodial meristems (SYMs). These SYMs generate two lateral branches, forming a distinct “V”-shaped branching pattern called a sympodial unit (SU). New SYMs repeatedly emerge from the axils of the uppermost leaves of each preceding SU, continuing the growth cycle [3]. In pepper (*C*. *annuum* L.), the angle between these two initial lateral branches is referred to as the first branch angle (FBA), which is closely related to density tolerance and yield, making it an important indicator for breeding an ideal plant architecture [4]. In cultivation, the ideal plant architecture is neither excessively spreading nor overly compact, as both extremes are detrimental to yield. Plants that are too compact tend to be more vulnerable to diseases, while those with a prostrate growth habit take up too much space, lowering the yield per unit area. Therefore, having the proper branch angle is not only beneficial for high-density planting but also promotes better ventilation and light penetration, enhances photosynthetic efficiency, reduces disease incidence, and improves the leaf area index and lodging resistance, ultimately increasing the overall yield [5–7]. Therefore, genetic analysis of the FBA trait and the identification of related genes are of great significance for improving pepper architecture [5, 6].

Current genetic research on branch, tiller, or leaf angle mainly focuses on plants such as *Arabidopsis thaliana*, rice, and oilseed rape [6–9]. In particular, genome-wide association studies (GWAS) have provided significant insights into the genetic architecture of these traits in major crops, leveraging natural genetic variation to identify candidate genes without the need for biparental populations. In rice, GWAS has been successfully applied to identify key loci controlling tiller angle, such as *TAC3*, *TAC1*, *D2*, *PAY1*, and *TIG1*, which are involved in gravitropism, brassinosteroid biosynthesis, strigolactone signaling, and cytoskeletal organization [10, 11]. In *Brassica napus*, GWAS has revealed multiple QTLs associated with branch angle, highlighting the polygenic nature of this trait [8, 12, 13]. These studies demonstrate the efficacy of GWAS in uncovering genetic determinants of plant architecture, providing a robust foundation for similar investigations in pepper. Beyond genetic factors, the branch, tiller, and leaf angles are also shaped by phytohormones as well as environmental cues such as light, gravity, and planting density [7]. Efforts to decipher the underlying regulatory mechanism have yielded substantial insights via cytological observations, hormonal detection, and transcriptome analyses across different species. Specifically, cytological observations have clarified key structural determinants, including the number of starch granules, the distribution of amyloplasts in the endodermis, and variations in xylem size [8, 14]; hormonal detection has identified critical phytohormones involved in the process, such as indole-3-acetic acid (IAA), gibberellins (GAs), cytokinins (CKs) and abscisic acid (ABA) [15–17]; transcriptome analyses have further unraveled the molecular pathways underlying these physiological and hormonal changes, covering plant hormone biosynthesis and metabolism, hormone signal transduction, and the regulation of cell division [15–18]. These collectively provide valuable methodological and theoretical references for investigating FBA in pepper.

It is noteworthy that, FBA in pepper, which has a sympodial shoot structure, differs from the following: the rosette branch angle in *A*. *thaliana*, referring to the angle between the cauline branch and inflorescence stem [19]; the branch angle in oilseed rape (*B. napus* L.), which refers to the angle between the branch base and the main stem [8]; the rice tiller angle, which is defined as the angle between the vertical line and the side tillers with maximum inclination [7]; and the leaf angle in maize, which refers to the divergence of the leaf blade from the main stem [20]. Notwithstanding these structural differences, some progress has been made in the study of FBA regulation in pepper. For instance, Liu [4] analyzed the genetic model of FBA in pepper based on the BB3×G-1 F_2_ population, concluding that its inheritance followed the E-1 model, specifically a two-major-gene additive–dominant–epistatic/polygenic additive–dominant genetic model, and mapped a major quantitative trait locus (QTL) to linkage group 12 at the 0–17.5 cM position, accounting for 5.1% of the phenotypic variation. Jiang [21] identified 17 FBA-related QTLs on chromosomes 1–10, accounting for 0.24–1.89% of the phenotypic variation, based on the F_2_ population. In addition, *CaVIL1*, a gene that promotes flowering in peppers, influences the angle of lateral branches [22].

However, the key genetic determinants and the underlying regulatory network controlling FBA in pepper remain largely unknown. To address this, we employed an integrated multi-omics approach. First, a GWAS was employed for the first time to investigate FBA in a natural pepper population, leveraging its powerful capability to exploit historical recombination events and uncover genetic associations without relying on bi-parental crosses. Then accessions with extreme phenotypes were selected to: (i) investigate the cytological basis of FBA variation; (ii) quantify the levels of major phytohormones; and (iii) conduct comparative transcriptomics to unravel the differentially regulated pathways and genes. This strategy allowed us to bridge the gap between genetic association and physiological function, providing a comprehensive understanding of FBA regulation in pepper.

## Materials and methods

### Plant accessions and phenotyping

In this study, 220 *C*. *annuum* accessions (Table S1), primarily sourced from 28 provinces across China, with a smaller proportion (10%) originating from other countries, were used for GWAS [23]. This collection, constituting 71.65% of a core collection [24], was further supplemented with additional breeding parents selected based on phenotypic and geographic data, as well as expert consultation. Field experiments were conducted in three different environments: Yichun, Jiangxi, China (115.13°E, 28.24°N) during the 2021 spring growing season (E1); Sanya, Hainan, China (109.08°E, 18.37°N) during the 2022 winter growing season (E2); and Yichun, Jiangxi, China (115.13°E, 28.24°N) during the 2023 winter growing season (E3). For each accession, twelve plants were grown at each experimental site under a randomized complete block design with three replications. All management followed standard local cultivation practices. Three plants in each replicate were selected to measure the FBA (the angle between the two initial lateral branches of the first sympodial unit) at the green-mature stage of the fruit located in the third layer of the sympodial shoot structure, which contains four sympodial units [25]. In addition, fruit traits (fruit shape index, fruit weight, and pericarp thickness), first flower node [26], main stem height, and plant height were measured in the three environments [23].

### GWAS for FBA in pepper

The 220 pepper accessions were genotyped using a genotyping-by-sequencing approach. After applying filtering criteria, 955,772 high-quality variants were identified, comprising 919,743 single nucleotide polymorphisms (SNPs) and 36,029 insertion–deletions (Indels) [23]. These variants were utilized in this study. Further details on the genotyping process and variant calling for the pepper accessions were previously reported [23]. Six models, namely a simple linear model (GLM), a generalized linear model (GLM(Q)), a mixed linear model (MLM(K)), a mixed linear model (MLM(QK)), bayesian-information and linkage-disequilibrium iteratively nested keyway (BLINK), and fixed and random model circulating probability unification (FarmCPU) were utilized for the association study using GEMMA [27] BLINK [28] and rMVP [29]. The best linear unbiased estimators (BLUEs) for FBA traits were calculated using the R packages ‘lme4’ and ‘lsmeans’. These FBA-BLUE values were subsequently applied in the GWAS analysis. Manhattan and quantile–quantile (QQ) plots for GWAS were generated with customized R script in conjuction with the ‘ggplot2’ package. The logarithm of the odds (LOD) threshold was adjusted using Bonferroni correction and defined as − log_10_ (1 × 10^−5^) to detect significant SNPs.

### Cytological observation of the first branch junctions

The first branch junctions were collected at the early flowering stages and fixed in formalin/acetic acid/alcohol (FAA) for 24 h. After serial dehydration, the samples were embedded in paraffin. Tissues were cut into 10 μm sections using a Leica microtome (RM2016; Leica, Wetzlar, Germany). The paraffin sections were deparaffinized and hydrated using a series of alcohol and liquid treatments, followed by rewarming, fixation, and Periodic Acid-Schiff (PAS) staining. The sections were then dehydrated, cleared with xylene, sealed with neutral gum, observed under a microscope (Eclipse E100; Nikon, Tokyo, Japan), and photographed with a digital microscope camera (DS-U3; Nikon, Tokyo, Japan). There were at least three biological replicates for each accession.

### Measurement of endogenous hormones

The contents of auxin precursors (indoleacetamide, IAM; indoleacetonitrile, IAN), auxin (IAA), conjugated auxin (indole-3-acetic acid asparagine, IAA-ASP), ABA, GAs (gibberellin A1, GA1; gibberellin A3, GA3; gibberellin A4, GA4), CKs (zeatin, ZT; trans-zeatin, tZ; isopentenyl adenine, iP), BRs (brassinolide, BL; castasterone, CS; 28-homobrassinolide, 28-HBL), and SLs (strigol; 5-deoxystrigol, 5-DS) were quantified using a high-performance liquid chromatography (HPLC) system (1290; Agilent, Palo Alto, CA, USA) coupled with a mass spectrometer (Qtrap 6500; AB SCIEX, Boston, MA, USA), referred to as HPLC-MS/MS. The first branch junctions were sampled at the early flowering stages for phytohormone content analysis. For BR and SL detection, branch junctions weighing 2–2.5 g were pooled into one sample, with 3 replicates per accession. For other hormones, 200–300 mg of branch junction tissues was pooled into 1 sample, with 3 replicates.

For BR detection, samples cryogenically pulverized in liquid nitrogen, followed by cold extraction with pre-chilled 95% methanol at 4 °C for 2 h. After centrifugation, the supernatant was purified using MCX columns, eluted with methanol, dried under nitrogen, reconstituted in methanol, filtered, and analyzed using HPLC-MS/MS. A Poroshell 120 SB-C18 reverse-phase column (Agilent, Palo Alto, CA, USA) was used at 35 °C, with a mobile phase of methanol and water/0.1% ammonium hydroxide, gradient elution, and a 2 µL injection volume. Mass spectrometry was performed in ESI positive ion mode using MRM scanning. The operating parameters were as follows: ionization voltage was set at + 4500 V, atomizing gas pressure was maintained at 65 psi, auxiliary gas pressure was adjusted to 70 psi, and the atomizing temperature was kept at 350 °C.

For SL detection, samples were ground in liquid nitrogen, extracted with pre-chilled acetone, ultrasonicated for 30 min, and extracted overnight at 4 °C. The supernatant was collected, dried under nitrogen, reconstituted, purified with HLB columns (Anpel Laboratory Technologies Inc., Shanghai, China), and analyzed using HPLC-MS/MS in acetonitrile. A Poroshell 120 SB-C18 reverse-phase column was used at 30 °C, with a mobile phase consisting of acetonitrile and water/0.1% formic acid, gradient elution, and a 2 µL injection volume. ESI positive ion mode was used with MRM scanning under the following conditions: ionization voltage of + 4500 V, atomizing gas pressure of 65 psi, auxiliary gas pressure of 70 psi, and atomizing temperature of 400 °C.

For the other hormones, samples were extracted with acetonitrile at a low temperature, purified using C18 and GCB sorbents (Anpel Laboratory Technologies Inc., Shanghai, China), dried under nitrogen, and reconstituted in methanol. The HPLC conditions were as follows: column temperature, 30 °C; gradient elution with methanol and water; flow rate, 0.3 mL/min; and 2 µL injection volume. Mass spectrometry was performed with the following settings: ESI ionization in both positive and negative ion modes; MRM scan mode; spray voltage, ± 4500 V; nebulizer gas pressure, 65 psi; and nebulizer temperature, 400 °C. Standard curves were established using phytohormone standards (Sigma, St. Louis, MO, USA).

### RNA sequencing and transcriptome analysis

Tissue samples were collected from B010 and B003 at the first branch sites during the early flowering stages for RNA-sequencing (RNA-seq) analysis. Tissue samples from eight individual plants were pooled to form a single biological replicate, with three replicates for each accession. Total RNA was isolated, and the cDNA libraries were constructed and sequenced using an Illumina NovaSeq X Plus platform (Illumina, San Diego, CA, USA) based on the PE150 strategy. The raw data were quality controlled using Fastq (v0.20.0) [30] with the following parameters: -q 5 -n 5. The filtered data were mapped to the reference genome *C*. *annuum* cv.59 (PRJNA788020) using HISAT2 (v2.1.0) [31]. Transcript abundance was quantified using fragments per kilobase of exon per million mapped reads (FPKM). Differentially expressed genes (DEGs) were considered to be those with |FoldChange| ≥ 1 and p-adjust (false discovery rate, FDR) ≤ 0.05. Differentially expressed genes were annotated using Blastall software (v2.2.26) against several databases, namely Gene Ontology (GO), Kyoto Encyclopedia of Genes and Genomes (KEGG), Clusters of Orthologous Groups (COG), Eukaryotic Orthologous Groups (KOG), Non-Redundant (NR), Swiss-Prot, and Pfam, to analyze their functions.

### Quantitative reverse transcription PCR (qRT-PCR)

qRT-PCR was performed as previously described to confirm the relative transcription levels of selected genes [26]. Briefly, total RNA was converted into cDNA using TransScript^®^ II One-Step gDNA Removal and cDNA Synthesis SuperMix (TransGen Biotech, Beijing, China). Subsequently, qRT-PCR was carried out on a Bio-Rad CFX Duet Real-Time PCR System (Bio-Rad Laboratories, Hercules, CA, USA) with NovoStart^®^ SYBR qPCR SuperMix Plus (Novoprotein Scientific Inc., Shanghai, China). Expression levels were normalized to *CaGAPDH* and calculated using the 2^−ΔΔCT^ method [32]. The primer sequences utilized for qRT-PCR analysis are detailed in Table S2, and the primer sequences for *CaGAPDH* were reported in a previous study [26]. Each sample was analyzed with triplicate technical replicates and three independent biological replicates.

## Results

### Phenotypic variation of FBA in pepper

**Fig. 1:**
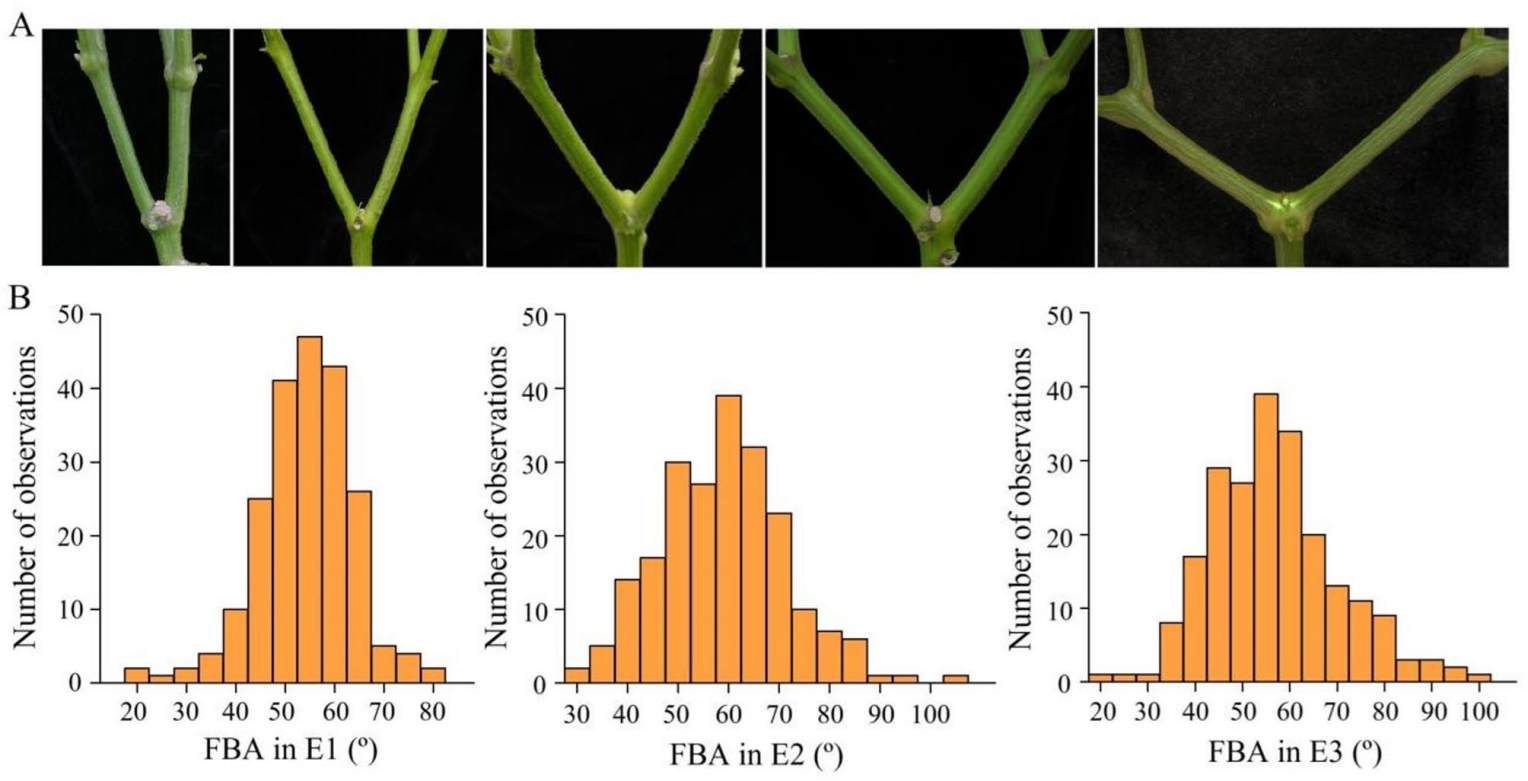
Variation in the first branch angle (FBA) of pepper. **A** Representative examples of pepper plants with different FBAs. **B** Frequency distribution of FBA among 220 pepper accessions grown in 3 environments: Yichun-2021-Spring (E1), Sanya-2022-Winter (E2), and Yichun-2023-Winter (E3)

To explore the phenotypic variation of FBA in a natural pepper population, FBA was evaluated across three environments. Genetic variation was observed among the 220 pepper accessions (Fig. 1A), with FBA ranging from 18.12° to 80.48° in E1, 32.08° to 102.52° in E2, and 21.74° to 97.85° in E3. The corresponding coefficients of variation (CV) were 17.83%, 21.22%, and 23.55% (Table 1). The FBA of the 220 accessions was normally distributed in E1 and E2 (*p* > 0.05) but not in E3 (*p* < 0.05) (Fig. 1B), suggesting that the low temperature and weak light conditions in E3, which differed from the conditions in E1 and E2, had affected the FBA trait. Broad-sense heritability (H²) was determined to be 93.26%, while narrow-sense heritability (h^2^) reached 72.06%. Based on FBA and overall performance, accessions with a moderately compact plant architecture, including B420, B430, B436, B446, C001, C032, C034, C153, C167, C189, WY003, WY035, WY119, WY167, WY229, WY248, WY260, WY284, WY291, and ZHE10, can provide breeding materials for developing pepper varieties with ideal plant types.

**Table 1:** Phenotypic data for the first branch angle in 220 pepper accessions

Environment	Min	Max	Mean ± SD	CV (%)	Kurtosis	Skewness
E1	18.12	80.48	54.11 ± 9.65	17.83	1.57	−0.52
E2	32.08	102.52	58.95 ± 12.51	21.22	0.32	0.38
E3	21.74	97.85	56.62 ± 13.34	23.55	0.46	0.54

*Min* minimum, *Max* maximum, *SD* standard deviation, *CV* coefficient of variation; E1, Yichun-2021-Spring; E2, Sanya-2022-Winter; E3, Yichun-2023-Winter

### Genome‑wide association study of FBA in pepper

**Fig. 2:**
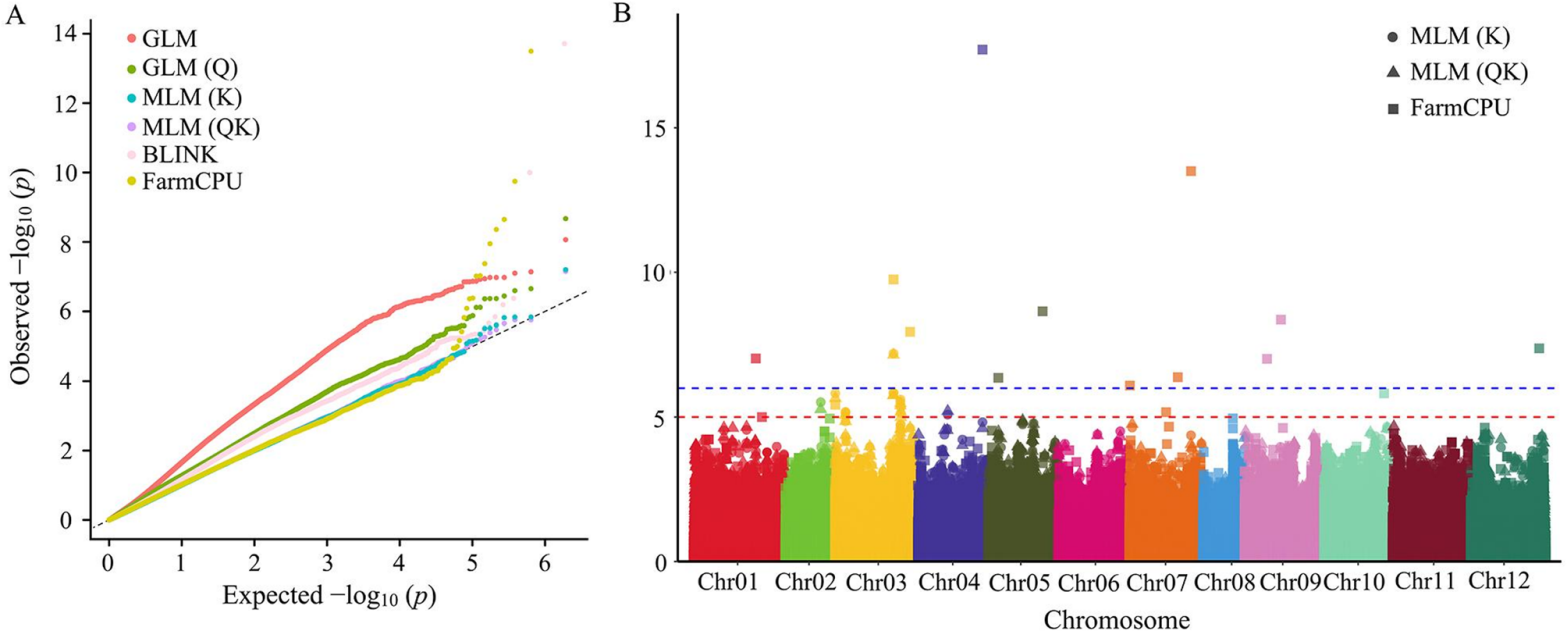
Genome-wide association study (GWAS) for the first branch angle (FBA) of 220 pepper accessions. **A** Quantile–quantile (QQ) plot of the six models. **B** Manhattan plot of GWAS for FBA among all chromosomes based on mixed linear model (MLM) and FarmCPU. The blue dashed lines corresponds to the statistical threshold of –log (1 × 10^−6^) and the red dashed line indicates –log (1 × 10^−5^). Circular symbols denote the MLM (K) results; triangular symbols denote the MLM (QK) results; square symbols denote the FarmCPU results

We performed a genome-wide association study of a panel of 220 accessions to explore the genetic foundation of FBA in pepper. The BLUE values for FBA across the three environments were used in the GWAS analysis to reduce environmental variation, improve the accuracy of trait estimates, and increase the power to detect true genotype–trait associations. Association analysis for FBA was performed using the GLM, GLM (Q), MLM (K), MLM (QK), BLINK and FarmCPU models. The QQ plot of MLM (K), MLM (QK) and FarmCPU (Fig. 2A) (with genomic inflation factors λ of 0.9927, 0.9945, and 1.0353, respectively) demonstrated strong agreement between the predicted and empirical p-values, with subsequent divergence, suggesting effective model calibration, which led to the detection of SNPs associated with FBA. A total of 25 SNPs were identified to cross the LOD threshold of 5 in the Manhattan plot, with LOD corresponding to –log (*p* = 1 × 10^−5^) (Fig. 2B). These SNPs accounted for 4.64–27.23% of the phenotypic variation. A SNP situated at position 220,539,044 bp on chromosome 4 was found to account for the most phenotypic variance (27.23%). Two SNPs, Chr03-1032256 and Chr03-199351423, were detected by all three methods. Our analysis revealed a suite of candidate genes for FBA implicated in various key pathways, encompassing phytohormone signaling (auxin, gibberellin, ethylene, brassinosteroid), receptor-mediated signal perception, calcium-dependent kinase cascades, protein ubiquitination, transcriptional regulation, and cell wall modification (Table 2). This includes specific genes encoding proteins such as BRI1, indole-3-pyruvate monooxygenase YUCCA10, gibberellin 20 oxidases, auxin-responsive protein SAUR50, F-box protein, calcium-dependent protein kinases (CDPKs), and expansin-A3 (Table 2).

**Table 2:** SNPs are significantly related to the first branch angle based on the mixed linear model (MLM) and fixed and random model Circulating probability unification (FarmCPU)

SNP	Chr	Pos	Model	*P* value	PVE^a^ value	Candidate genes^b^	Predicted function
Chr01-208719103	Chr1	208,719,103	FarmCPU	9.33 × 10^−8^	9.45%	*CA.PGAv.1.6.scaffold410.1* (−0.71)	E3 ubiquitin-protein ligase-like
Chr01-228881442	Chr1	228,881,442	FarmCPU	1.00 × 10^−5^	11.34%	*CA.PGAv.1.6.scaffold185.39* (−0.52)	ABC transporter C family member 4
						*CA.PGAv.1.6.scaffold185.38* (−0.36)	ABC transporter C family member 4-like
						*CA.PGAv.1.6.scaffold185.28* (0.44)	Leucine-rich repeat receptor-like serine/threonine-protein kinase BAM3
Chr02-120686854	Chr2	120,686,854	MLM (K)	3.04 × 10^−6^	6.17%	*CA.PGAv.1.6.scaffold519.11* (1.05)	Calcium-dependent protein kinase 24-like
			MLM (QK)	5.39 × 10^−6^	6.01%		
Chr03-1032256	Chr3	1,032,256	MLM (K)	1.51 × 10^−6^	8.50%	*CA.PGAv.1.6.scaffold710.22* (0.17)	Gibberellin 20 oxidase 1-like
			MLM (QK)	2.21 × 10^−6^	8.45%		
			FarmCPU	3.80 × 10^−6^	10.24%		
Chr03-35861683	Chr3	35,861,683	MLM (K)	7.00 × 10^−6^	7.04%	*CA.PGAv.1.6.scaffold426.24* (1.06)	Probable indole-3-pyruvate monooxygenase YUCCA10
			MLM (QK)	8.08 × 10^−6^	6.99%		
Chr03-35866908	Chr3	35,866,908	MLM (K)	6.58 × 10^−6^	7.44%		
			MLM (QK)	8.68 × 10^−6^	7.35%		
Chr03-199351423	Chr3	199,351,423	MLM (K)	6.29 × 10^−8^	8.65%	*CA.PGAv.1.6.scaffold1717.1* (0.83)	Interactor of constitutive active ROPs 4-like
			MLM (QK)	7.04 × 10^−8^	8.73%		
			FarmCPU	1.78 × 10^−10^	17.99%	*CA.PGAv.1.6.scaffold48.42 (0.36)*	F-box protein
Chr03-199351456	Chr3	199,351,456	MLM (K)	1.46 × 10^−6^	6.37%	*CA.PGAv.1.6.scaffold48.45* (0.24)	BRI1 protein
			MLM (QK)	1.75 × 10^−6^	6.36%		
Chr03-199351464	Chr3	199,351,464	MLM (K)	1.46 × 10^−6^	6.37%	*CA.PGAv.1.6.scaffold48.42* (0.36)	F-box protein At5g51370
			MLM (QK)	1.75 × 10^−6^	6.36%	*CA.PGAv.1.6.scaffold48.33* (0.94)	Putative nitric oxide synthase
Chr03-223021763	Chr3	223,021,763	MLM (K)	2.46 × 10^−6^	7.81%	*CA.PGAv.1.6.scaffold548.24* (0.10)	Calcium-dependent protein kinase 26
			MLM (QK)	3.31 × 10^−6^	7.68%		
Chr03-223021839	Chr3	223,021,839	MLM (K)	2.98 × 10^−6^	7.70%	*CA.PGAv.1.6.scaffold548.21* (0.12)	ABC transporter B family member 28
			MLM (QK)	4.01 × 10^−6^	7.56%		
Chr03-224443849	Chr3	224,443,849	MLM (K)	4.50 × 10^−6^	6.42%	-	-
			MLM (QK)	6.69 × 10^−6^	6.26%		
Chr03-224478723	Chr3	224,478,723	MLM (K)	7.23 × 10^−6^	4.64%	-	-
Chr03-256330769	Chr3	256,330,769	FarmCPU	1.12 × 10^−8^	11.07%	*CA.PGAv.1.6.scaffold637.53* (0.37)	Transcription factor bHLH137-like
						*CA.PGAv.1.6.scaffold637.42* (0.56)	Mitogen-activated protein kinase kinase 9
						*CA.PGAv.1.6.scaffold637.30* (0.87)	Calmodulin
Chr04-101827672	Chr4	101,827,672	MLM (K)	8.25 × 10^−6^	6.06%	-	-
			MLM (QK)	6.21 × 10^−6^	6.21%		
Chr04-220539044	Chr4	220,539,044	FarmCPU	1.95 × 10^−18^	27.23%	*CA.PGAv.1.6.scaffold1658.8* (0.08)	Transcription factor bHLH143
						*CA.PGAv.1.6.scaffold1697.8* (0.46)	Serine/threonine-protein kinase-like protein
						*CA.PGAv.1.6.scaffold1375.11* (0.85)	F-box/kelch-repeat protein
Chr05-34719330	Chr5	34,719,330	FarmCPU	4.37 × 10^−7^	13.60%	*CA.PGAv.1.6.scaffold74.16* (0.50)	Microtubule-associated protein 70 − 2
Chr05-185210660	Chr5	185,210,660	FarmCPU	2.24 × 10^−9^	10.91%	*CA.PGAv.1.6.scaffold1153.8* (−0.88)	Pectinesterase/pectinesterase inhibitor 12
						*CA.PGAv.1.6.scaffold112.1* (−0.11)	Ethylene-response factor C3
						*CA.PGAv.1.6.scaffold112.*2 (0.02)	Ethylene-responsive transcription factor C3
Chr07-2793550	Chr7	2,793,550	FarmCPU	8.13 × 10^−7^	11.67%	*CA.PGAv.1.6.scaffold584.17* (−0.43)	Transcription factor MYB36
						*CA.PGAv.1.6.scaffold584.27* (−0.27)	Receptor-like serine/threonine-protein kinase
						*CA.PGAv.1.6.scaffold584.28* (−0.25)	PTI1-like tyrosine-protein kinase
Chr07-125936312	Chr7	125,936,312	FarmCPU	6.76 × 10^−6^	9.22%	*CA.PGAv.1.6.scaffold212.4* (0.00)	Polygalacturonase-like
Chr07-165805490	Chr7	165,805,490	FarmCPU	4.17 × 10^−7^	8.03%	*CA.PGAv.1.6.scaffold81.5* (−0.49)	Plasma membrane ATPase 4
Chr07-210841642	Chr7	210,841,642	FarmCPU	3.16 × 10^−14^	21.29%	*CA.PGAv.1.6.scaffold1740.1* (0.06)	Beta-1,3-galactosyltransferase 2
Chr09-124248226	Chr9	124,248,226	FarmCPU	4.37 × 10^−9^	12.93%	*CA.PGAv.1.6.scaffold708.2* (−0.73)	Gibberellin 20 oxidase 3-like
Chr10-204614298	Chr10	204,614,298	FarmCPU	1.51 × 10^−6^	11.64%	*CA.PGAv.1.6.scaffold324.9* (−0.53)	Expansin-A3
Chr12-233823353	Chr12	233,823,353	FarmCPU	4.27 × 10^−8^	11.56%	*CA.PGAv.1.6.scaffold847.14* (0.54)	Auxin-responsive protein SAUR50-like
						*CA.PGAv.1.6.scaffold847.15* (0.50)	Auxin-responsive protein SAUR50-like

^a^ Percent variance explained; ^b^ The numbers in parentheses indicate the physical distances (Mb) between candidate genes and SNPs

### Divergence of FBA among the four clusters

**Fig. 3:**
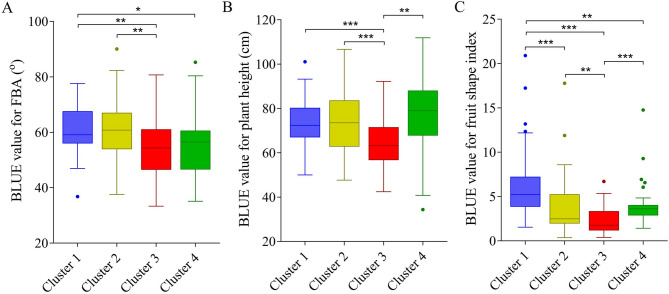
Divergence of the first branch angle (FBA) in the four pepper clusters. **A** Box plot showing the best linear unbiased estimator (BLUE) values for FBA in the four pepper clusters. Scale bar: 5 cm. **B** Box plot showing the BLUE values for plant height in the four pepper clusters. **C** Box plot showing the BLUE values for fruit shape index in the four pepper clusters. Significance of differences between clusters or genotypes: * represents *p* < 0.05, ** represents *p* < 0.01, *** represents *p* < 0.001

Based on our previous research, the 220 accessions for GWAS were categorized into 4 distinct clusters. Cluster 4, an ancestral group, features small fruit, late maturity, and high genetic diversity. By contrast, cluster 2 is a transitional cluster with diverse fruit shapes and exhibits high genetic diversity. Clusters 1 and 3 are characterized by a long fruit shape and large-fruited peppers, respectively, demonstrating lower genetic diversity and higher linkage disequilibrium, indicating recent selective breeding [23]. The FBA values in these four clusters were calculated and compared. The FBA values of the large-fruited cluster 3 (54.27 ± 1.24°) and ancestral cluster 4 (55.30 ± 1.69°) were lower, while the FBA values of the long fruit-shaped cluster 1 (60.69 ± 1.14°) and transitional cluster 2 (60.15° ± 1.36°) were higher. Significant differences in FBA values were observed between clusters 1 and 4 (*p* < 0.05) as well as between cluster 3 and both clusters 1 and 2 (*p* < 0.01) (Fig. 3A).

To identify potential relationships between FBA and other traits, correlations were evaluated with plant height, main stem height, fruit shape index, fruit weight, pericarp thickness, and first flower node. A significant negative correlation (*p* < 0.01) was found between FBA and plant height, whereas a significant positive correlation (*p* < 0.05) was detected between FBA and the fruit shape index. In addition, cluster 3 exhibited a significantly lower plant height compared with the other three clusters (Fig. 3B). Significant differences in the fruit shape index were observed between each pair of clusters, except between clusters 2 and 4 (Fig. 3C). These findings suggest that the loci regulating FBA, plant height, and fruit shape index either overlap or are closely linked.

### Cytological observation of the first branch among accessions with divergent angles

**Fig. 4:**
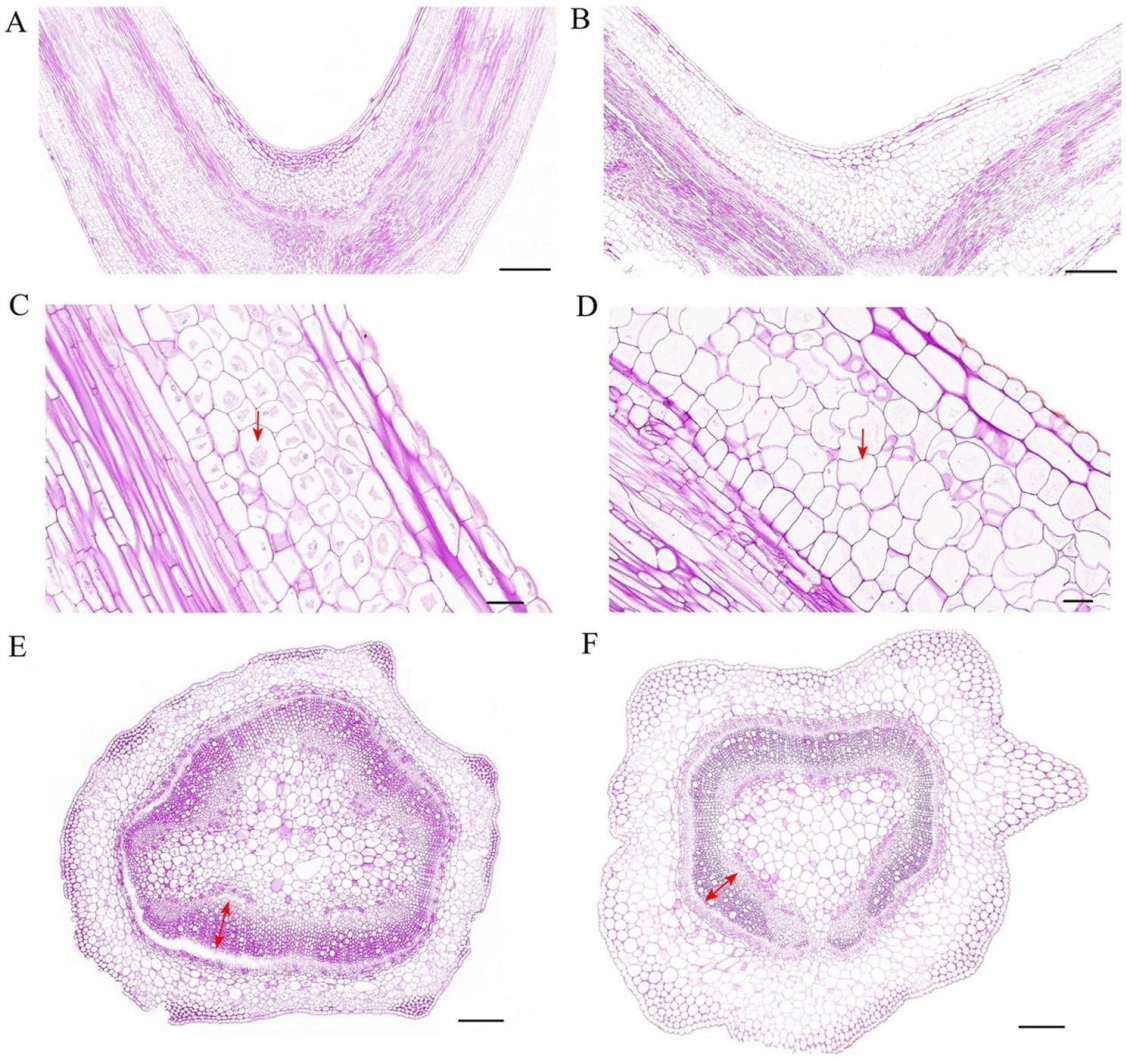
Cytological analysis of branch junctions in B010 and B003. **A** Longitudinal section of the branch junction in compact accession B010. **B** Longitudinal section of the branch junction in loose accession B003. **C** Magnified view of the endodermis in compact accession B010. **D** Magnified view of the endodermis in loose accession B003. **E** Horizontal section at the base of the branch junction in compact accession B010. **F** Horizontal section at the base of the branch junction in loose accession B003. Scale bars = 500 μm in (**A**, **B**), 50 μm in (**C**, **D**), 300 μm in (**E**, **F**). Red arrowheads: starch granules within endodermal amyloplasts in (**C**, **D**). Red double-headed arrows: xylem in (**E**, **F**)

Based on the GWAS results that identified candidate genes involved in cell wall modification (e.g., Expansin-A3), we hypothesized that differences in branch angle architecture may be underpinned by variations in cellular morphology. To investigate the cytological basis for branch angle differences, paraffin sections of the branch junctions from accessions B010 (which had a small branch angle) and B003 (which had a large branch angle) were examined under a microscope (Fig. 4). The paraffin sections were stained with PAS to identify starch-filled amyloplasts, specialized organelles known to sense gravity and help plants orient their growth. The longitudinal sections showed that compact accession B010 contained more starch granules in endodermal amyloplasts (Fig. 4A and C) than those in the loose accession B003 (Fig. 4B and D). The cell diameters in the epidermis and endodermis on the adaxial side of the loose accession (86.89 ± 2.14 and 51.67 ± 2.39 μm, respectively) were 39.92% and 135.94% larger, respectively (both *p* < 0.01), compared with those in the compact accession (62.10 ± 1.94 and 21.90 ± 1.03 μm, respectively) (Fig. 4B and D). Furthermore, a horizontal cross-section at the branch–stem junction revealed that the vascular bundle of B010 exhibited a larger xylem (Fig. 4E) compared with B003 (Fig. 4F).

### Hormone content of the first branch in accessions with divergent angles

**Fig. 5:**
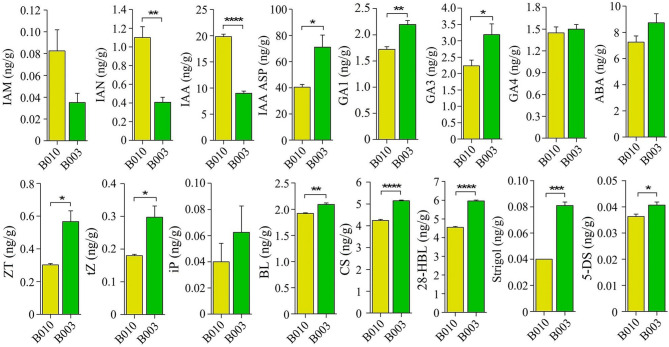
The contents of 12 hormones in the first branches of compact accession B010 and loose accession B003. Asterisks show significant differences between B010 and B003 (Student’s t-test, * *p* < 0.05, ** *p* < 0.01, *** *p* < 0.001, **** *p* < 0.0001). IAM: indoleacetamide; IAN: indoleacetonitrile; IAA: indole-3-acetic acid; IAA ASP: indole-3-acetic acid asparagine; GA1: gibberellin A1; GA3: gibberellin A3; GA4: gibberellin A4; ABA: abscisic acid; ZT: zeatin; tZ: trans-zeatin; iP: isopentenyl adenine; BL: brassinolide; CS: castasterone; 28-HBL: 28-homobrassinolide; 5-DS: 5-deoxystrigol

Given that the GWAS candidate genes included hormone-related genes (e.g., *CA*.*PGAv*.*1.6.scaffold48*.*45*, *CA*.*PGAv*.*1.6*.*scaffold710*.*22*, *CA*.*PGAv*.*1*.*6*.*scaffold426*.*24*, *CA.PGAv.1.6.scaffold708.2*, and *CA.PGAv.1.6.scaffold847.14*), we hypothesized that phytohormone is crucial for FBA regulation. To verify this, the hormone contents of the first branch in accessions with divergent angles were determined using HPLC-MS/MS. The contents of auxin precursors (IAN and IAM) and auxin (IAA) in compact accession B010 were significantly higher than those in loose accession B003, with the differences in IAN and IAA reaching statistical significance (*p* < 0.01; *p* < 0.0001) (Fig. 5). By contrast, the conjugated auxin (IAA ASP) content in B010 was significantly lower than in B003 (*p* < 0.05). In addition, the GA (GA1, GA3, and GA4), CK (ZT, tZ, and iP), ABA, BR (BL, CS, and 28HBL), and SL (Strigol and 5-DS) levels were all lower in B010 than in B003 (Fig. 5). The differences in GA1 (*p* < 0.01), GA3 (*p* < 0.05), zeatin (*p* < 0.05), tZ (*p* < 0.05), BL (*p* < 0.01), CS (*p* < 0.0001), 28-HBL (*p* < 0.0001), strigol (*p* < 0.001), and 5-DS (*p* < 0.05) were statistically significant, whereas the differences in GA4, iP, and ABA were not significant (Fig. 5).

### Comparative transcriptomic analysis of the first branches

**Fig. 6:**
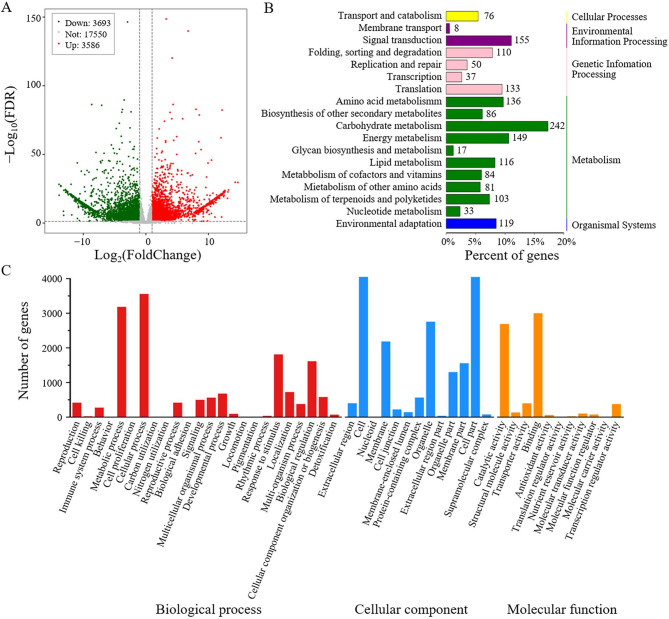
Differentially expressed genes (DEGs) between compact accession B010 and loose accession B003. **A** Volcano plot depicting the significantly upregulated and downregulated genes between B010 and B003. **B** KEGG pathway analysis of DEGs. **C** Functional categorization of DEGs between B010 and B003 according to Gene Ontology (GO) analysis

Building on the genetic loci identified by GWAS and the observed hormonal differences between accessions, we performed a comparative transcriptomic analysis to further elucidate the molecular mechanisms underlying branch angle divergence. At the early flowering stage, the first branch sites of B010 and B003 were sampled for RNA sequencing, with three biological replicates for each accession. After removing low-quality sequences, 80,049,780 and 73,010,958 pair-end clean reads were obtained from B010 and B003, respectively, and 95.24% and 94.82% of reads, respectively, were successfully mapped to the *C*. *annuum* cv.59 reference genome. Principal component analysis (PCA) revealed that the B010 samples clustered separately from the B003 samples, with clear differentiation along the PC1 and PC2 axes, indicating variation between these accessions (Fig. S1). A total of 7,279 DEGs were identified in the comparison between compact accession B010 and loose accession B003. Among these, 3,586 DEGs were consistently upregulated in B010 compared with B003, while 3,693 DEGs were downregulated in B003 (Fig. 6A). GO classification analysis showed that the majority of DEGs were primarily involved in biological processes such as cellular and metabolic processes, cellular components such as the cell and cell part, and molecular functions related to binding and catalytic activity (Fig. 6C). According to KEGG analysis, the DEGs were mainly involved in carbohydrate metabolism and signal transduction (Fig. 6B).

**Fig. 7:**
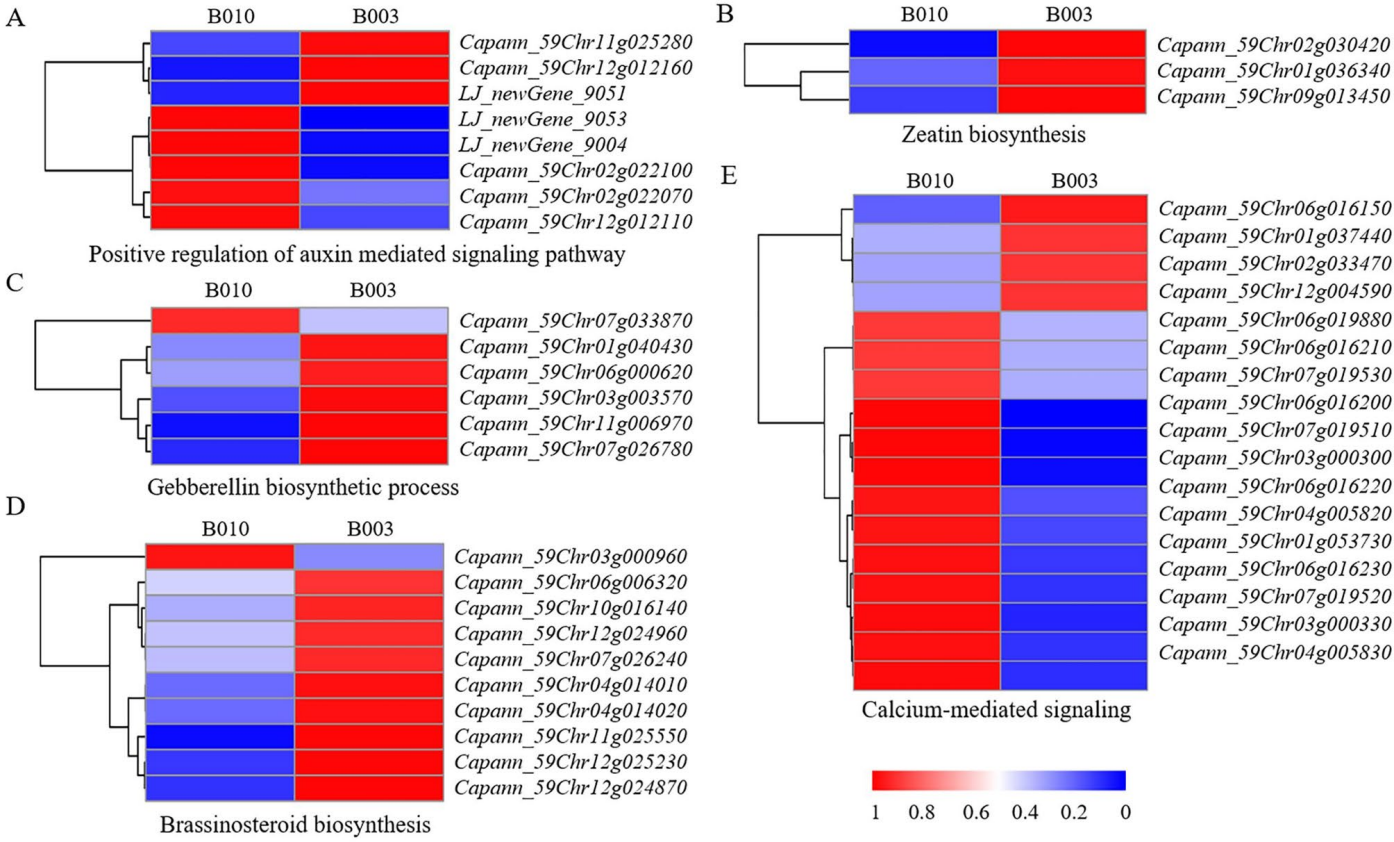
Heat maps of differentially expressed genes (DEGs) associated with branch angle. **A** DEGs related to the positive regulation of the auxin-mediated signaling pathway. **B** DEGs participating in zeatin biosynthesis. **C** DEGs contributing to the gibberellin biosynthesis process. **D** DEGs engaged in brassinosteroid biosynthesis. **E** DEGs implicated in calcium-mediated signaling. The color scale represents standardized fragments per kilobase of exon per million fragments mapped (FPKM) values, with blue indicating low expression and red representing high expression

DEGs were significantly enriched in several biological processes associated with the GWAS candidate genes as well as the differential hormones between B010 and B003, such as the positive regulation of the auxin-mediated signaling pathway (Fig. 7A) and calcium-mediated signaling (Fig. 7E), in which most DEGs were downregulated in B003, zeatin biosynthesis (Fig. 7B), gibberellin biosynthesis (Fig. 7C), and brassinosteroid biosynthesis (Fig. 7D), in which most DEGs were upregulated in B003. Furthermore, two candidate genes identified through GWAS analysis, namely *CA.PGAv.1.6.scaffold1697.8* (*Capann_59Chr04g009940*) and *CA.PGAv.1.6.scaffold584.27* (*Capann_59Chr07g001410*), were also found to be differentially expressed in the transcriptome data, suggesting their potential regulatory roles in FBA formation.

### qRT-PCR verification of DEGS related to branching

**Fig. 8:**
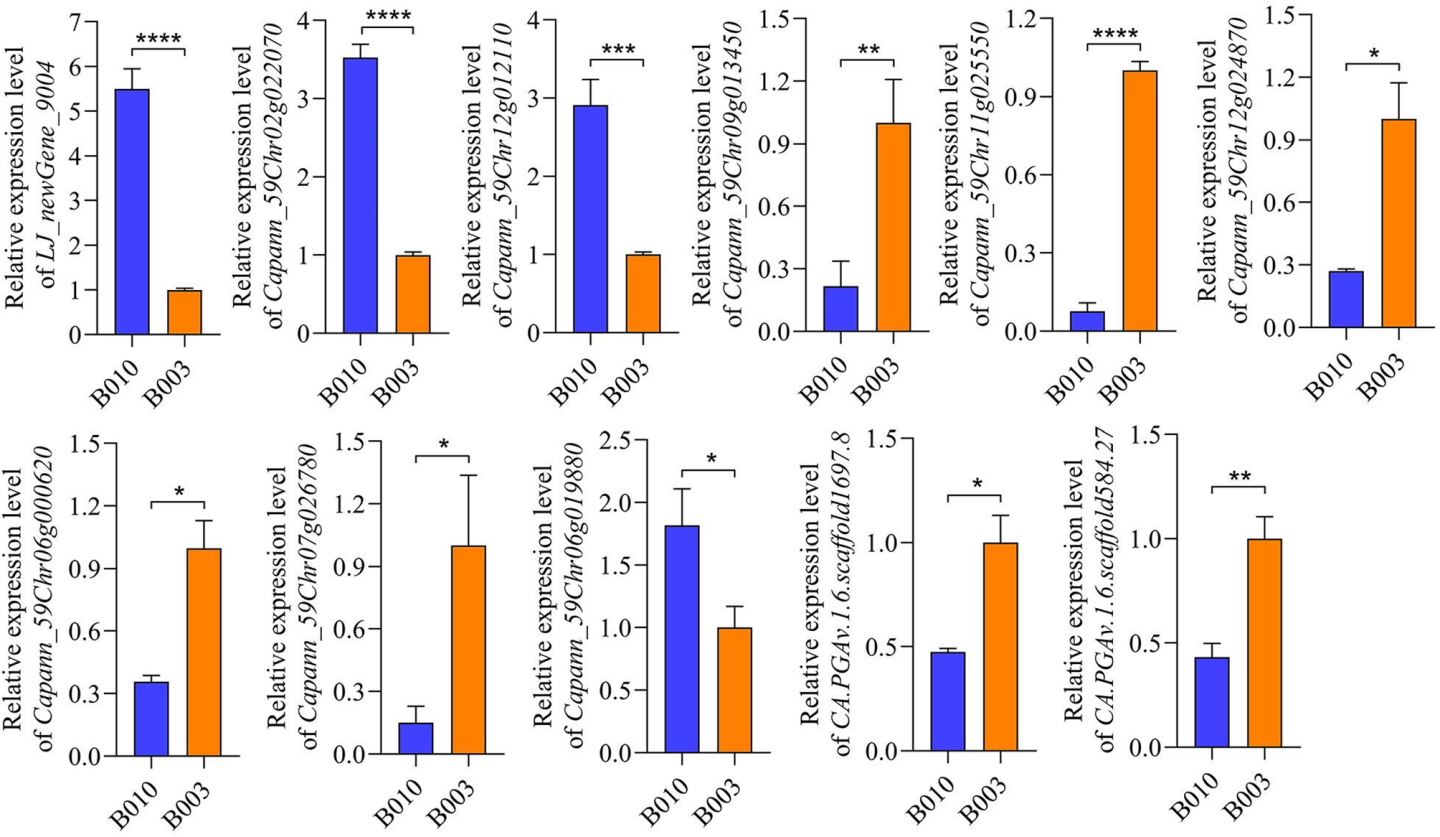
Relative gene expression levels of selected differentially expressed genes (DEGs) in B010 and B003 verified using qRT-PCR. Asterisks show significant differences between B010 and B003 (Student’s t-test, * indicates *p* < 0.05, ** represents *p* < 0.01, *** indicates *p* < 0.001, **** represents *p* < 0.0001)

To validate the transcriptome results, the expression levels of nine selected DEGs were analyzed using qRT-PCR. These DEGs includes seven associated with key pathways, such as positive regulation of auxin-mediated signaling, zeatin biosynthesis, gibberellin biosynthesis, brassinosteroid biosynthesis, and calcium-mediated signaling, as well as two candidate genes identified from the GWAS analysis (*CA.PGAv.1.6.scaffold1697.8* and *CA.PGAv.1.6.scaffold584.27*). Although differences in absolute expression levels were observed between RNA-seq and qRT-PCR, the expression trends were consistent across the selected genes. For example, *LJ_newGene_9004*, *Capann_59Chr02g022070*, and *Capann_59Chr12g012110*, which have been implicated in the positive regulation of auxin-mediated signaling, were upregulated in compact accession B010 compared with B003. By contrast, *Capann_59Chr09g013450* was downregulated in B010 relative to B003. Similarly, the GWAS candidate genes *CA.PGAv.1.6.scaffold1697.8* and *CA.PGAv.1.6.scaffold584.27* were significantly upregulated in B003 (Fig. 8). These results not only confirm the reliability of the transcriptome data, but also provide expression-level validation to support the genetic association identified in GWAS.

## Discussions

Current genetic research on branch, tiller, and leaf angles primarily focuses on plants such as *Arabidopsis*, rice, and oilseed rape, providing a foundational reference for this study. However, studies on FBA remain limited in pepper. In this study, we employed a combination of GWAS, cytological observations, hormone detection, and comparative transcriptomic analysis to determine the mechanisms underlying the FBA trait in pepper. The relatively high broad-sense heritability (H²) of 93.26% and narrow-sense heritability (h²) of 72.06% indicate that FBA is predominantly governed by genetic factors, with a relatively low influence from environmental conditions. This suggests that, despite observed environmental effects, the trait has strong genetic stability, making it an ideal target for selective pepper breeding programs.

### Candidate genes in associated loci for FBA traits

The GWAS employed in this study enables high-resolution dissection of the FBA trait in pepper by harnessing natural genetic variation among diverse germplasm resources, efficiently revealing its polygenic architecture and key genetic determinants. The Manhattan plot for MLM (K), MLM (QK), and FarmCPU identified 25 SNPs associated with FBA. The SNP located at position 199,351,423 bp on chromosome 3 was identified by all these three models, explaining up to 17.99% of the phenotypic variance in the FarmCPU model. Two additional associated SNPs, chr03-199,351,456 and chr03-199,351,464 identified by MLM (K) and MLM (QK), proximal to this SNP, further support the reliability of this locus as a key regulator of FBA. Genes surrounding this locus, including the interactor of constitutively active ROPs 4-like gene (*CA.PGAv.1.6.scaffold1717.1*), BRI1 protein-encoding gene (*CA.PGAv.1.6.scaffold48.45*), F-box protein At5g51370-encoding gene (*CA.PGAv.1.6.scaffold48.42*), and putative nitric oxide synthase-encoding gene (*CA.PGAv.1.6.scaffold48.33*), were considered potential candidate genes. Among these candidates, BRI1 protein-encoding gene (*CA.PGAv.1.6.scaffold48.45*) is considered a key candidate gene at this locus due to its close physical proximity to the associated SNP (0.24 Mb) and its functional relevance, as its rice homolog *OsBRI1*, a brassinosteroid receptor kinase regulated by OsARF19, is a known determinant of leaf angle architecture [33].

The Gibberellin 20 oxidase 1-like gene (*CA.PGAv.1.6.scaffold710.22*) was proposed as a candidate for controlling FBA. Firstly, a nearby SNP (chr03-1032256, located just 0.17 Mb away) was identified by all three models and explained 10.24% of the phenotypic variance in the FarmCPU model. Secondly, the predicted role of this gene in GA biosynthesis aligns with the established mechanism through which GAs modulate leaf inclination by promoting adaxial parenchyma cell proliferation and radial expansion in the leaf collar zone [34, 35].

In the FarmCPU model, the SNP Chr04-220539044 explained the highest proportion of phenotypic variance, with a substantial contribution of 27.23%. Two candidate genes were identified at this locus: *CA.PGAv.1.6.scaffold1658.8*, which encodes a bHLH143 transcription factor, and *CA.PGAv.1.6.scaffold1697.8*, which encodes a serine/threonine-protein kinase-like protein. Notably, *CA.PGAv.1.6.scaffold1697.8* emerged as a particularly promising candidate, not only due to the putative functional role of serine/threonine-protein kinase in auxin transport [36, 37] but also because its differential expression between the compact B010 and the loose B003. Similarly, a receptor-like serine/threonine-protein kinase-encoding gene, *CA.PGAv.1.6.scaffold584.27*, was identified as a putative candidate for SNP Chr07-2793550 (11.67% phenotypic variance explained in FarmCPU). Like the aforementioned candidate, this gene also showed differential expression between B010 and B003. Moreover, receptor-like serine/threonine-protein kinases are known to positively regulate brassinosteroid (BR) signaling [38], which provides functional relevance to its potential role in FBA.

In addition, *CA.PGAv.1.6.scaffold519.11*, a calcium-dependent protein kinase 24-like (CDPK24) gene located 1.05 Mb away from this Chr02-120686854, is a possible candidate given the involvement of CDPKs in phytohormone signaling pathways [39]. *CA.PGAv.1.6.scaffold548.24* (calcium-dependent protein kinase 26) and *CA.PGAv.1.6.scaffold548.21* (ABC transporter B family member 28) may represent candidate genes for the Chr03-223021763/Chr03-223021839 locus. ABCBs are necessary for polar auxin transport, suggesting their involvement at these loci [40, 41]. *CA.PGAv.1.6.scaffold426.24*, a probable indole-3-pyruvate monooxygenase YUCCA10 gene involved in auxin biosynthesis [42, 43], may serve as a candidate gene for the Chr03-35861683/Chr03-35866908 locus, as auxin regulates branch angle and gravitropic responses [44]. Given that SAUR proteins regulate branch angle by promoting auxin-mediated asymmetric cell elongation on the abaxial side, we propose that *CA.PGAv.1.6.scaffold847.14* and *CA.PGAv.1.6.scaffold847.15* (which encode an auxin-responsive SAUR50 protein) are the putative underlying genes for the locus Chr12-233823353. However, these SNPs require additional confirmation due to the presence of only one significantly associated SNP at their loci.

The identification of these candidate genes is of great significance for advancing FBA research in pepper, as they reveal novel genetic factors regulating this important trait and offer potential targets for breeding programs aiming to optimize pepper architecture. However, most of the candidate genes identified through GWAS did not show significant differential expression in the transcriptomic analysis between compact B010 and loose B003. Nevertheless, we observed an enrichment of the biological processes associated with these candidate genes, for example, brassinosteroid biosynthesis related to *BRI1*, gibberellin biosynthesis related to *Gibberellin 20 oxidase 1*, calcium-mediated signaling related to *CDPK24*, and auxin-mediated signaling pathways related to *YUCCA10*. This suggests that these pathways play an important role in regulating FBA in pepper. These genes might not regulate downstream biological processes via expression level differences but rather through variations in gene sequences or epigenetic modifications. Furthermore, since the candidate genes in this study were identified based on 220 pepper accessions, these genes might not be specifically related to the FBA differences observed between the two randomly chosen germplasm resources, B010 and B003.

### FBA variation and its relationship with key agronomic traits

This study revealed that FBA showed no significant changes from the ancestral group (cluster 4) to the transitional cluster (cluster 2). From cluster 2 to the recently selected long fruit-shaped cluster (cluster 1), FBA slightly decreased, but the reduction was not statistically significant. By contrast, a significant decrease in FBA was observed from cluster 2 to the recently selected large-fruited cluster (cluster 3). Notably, cluster 3 exhibited a significantly lower plant height and a smaller fruit shape index compared with the other three clusters. Previous research has shown that peppers with smaller branching angles tend to have greater plant height [21]. Therefore, the significant decrease in FBA from clusters 2 to 3 was likely a byproduct of selection for fruit shape (fruit shape index) or plant height. From a developmental perspective, shorter plants may allocate more resources to lateral growth, resulting in larger branch angles, whereas taller plants might invest more in vertical growth, limiting branch angle development.

Historically, selection in pepper breeding has primarily focused on fruit-related traits, such as fruit size, shape, and pungency [45]. However, with the increasing adoption of mechanized production in agriculture, there is a growing need to develop pepper varieties with a moderately compact plant architecture, higher planting density, reduced branch entanglement, and improved harvest efficiency. The genetic loci associated with FBA and the accessions with a moderately compact architecture (e.g., B420 and WY291) identified in this study provide a valuable foundation for future breeding efforts aiming to optimize pepper plant architecture.

### Cytological basis of FBA variation between divergent materials

To uncover the cellular basis for the substantial difference in branch angles between B010 and B003, we analyzed their branch junctions using paraffin section microscopy. The longitudinal sections revealed a higher number of amyloplasts within the endodermis of the compact accession B010 compared with the looser B003, suggesting that B010 exhibits greater responsiveness to gravity. The vascular bundle of B010 exhibited a larger xylem, suggesting that variation in xylem size could explain the difference in branch angles between compact and loose accessions. These findings align with previous studies in rapeseed and rice [8, 14] that made similar observations. Furthermore, the larger cell diameter observed in the loose accession suggests that the increased cell size in the junction area of the first branch contributes to greater separation between the two branches, thereby resulting in a wider branch angle, which is consistent with previous observations in rapeseed [46]. This observed significant increase in cell diameter is consistent with the proposed role of the candidate genes identified in GWAS, wherein the cell wall-loosening activity of expansin-A3 (*CA.PGAv.1.6.scaffold324.9*) likely promotes expansion, while the coordinated actions of polygalacturonase (*CA.PGAv.1.6.scaffold212.4*; pectin degradation), plasma membrane ATPase 4 (*CA.PGAv.1.6.scaffold81.5*; cell wall acidification), and β−1,3-galactosyltransferase 2 (*CA.PGAv.1.6.scaffold1740.1*; cell wall polysaccharide biosynthesis) are predicted to remodel the cell wall to facilitate this growth and govern the resultant branch angle.

### Mechanisms regulating FBA in pepper revealed by hormonal and transcriptomic profiling

Since hormones play a key role in regulating branch, tiller, and leaf angles, we focused on the differences in hormone levels between B010 and B003. Compact accession B010 showed significantly higher levels of auxin precursors (IAN) and free auxin (IAA) but a lower conjugated auxin (IAA-ASP) content compared with loose accession B003, consistent with IAA’s role as a negative regulator of leaf angle in corn, rice, and potato [47–50]. Correspondingly, the DEGs were significantly enriched in the positive regulation of the auxin-mediated signaling pathway, with most upregulated in the compact B010.

A relationship has been reported between CK and auxin, with CK acting downstream of auxin [51]. Auxin inhibits CK synthesis by repressing the expression of the *IPT* gene, which plays a key role in CK biosynthesis [52, 53]. This is consistent with our findings showing that the *Capann_59Chr01g036340* and *Capann_59Chr09g013450* genes, both annotated as IPT in KEGG and involved in ZT biosynthesis, were upregulated in B003. IPT is an essential enzyme for the synthesis of CKs, such as ZT, tZ, and iP [54]. In line with this, the levels of ZT, tZ, and iP in B003 were higher than those in B010, and the tZ and ZT levels showed a significant difference.

Previous studies have shown that GAs play a role in increasing the leaf angle by promoting the proliferation and expansion of parenchymal cells on the adaxial side of leaf joints [34, 55, 56]. Consistent with these findings, the GA1 concentration (the principal bioactive GA in rice) was significantly higher in B003 than in B010. This result was further supported by transcriptomic analysis, which revealed that DEGs were significantly enriched in GA biosynthesis, with the majority of DEGs upregulated in the loose B003.

BRs are synthesized from campesterol and converted into the biologically active compounds CS and BL [57, 58]. Among BRs, BL and CS exhibit high biological activity and are widely distributed in plants [59], whereas 28-HBL (which differs from BL at the C-24 position) has activity comparable to that of BL under certain conditions [60]. BR biosynthesis is crucial for controlling leaf angle since BRs stimulate cell elongation on the adaxial side of the lamina joint [61–64]. Defective/knockdown of BR biosynthesis enzyme genes reduced the rice tiller angle or caused erect leaves in rice [10, 62, 65–69]. In this study, we found that BL, CS, and 28-HBL levels were significantly higher in loose B003 than in compact B010. In addition, DEGs were enriched in the BR biosynthesis pathway, with most DEGs upregulated in B003. These results suggest that BRs play a key role in FBA regulation in pepper, likely by promoting cell elongation and contributing to larger cell size, as observed in loose B003.

SLs, a group of plant hormones biosynthesized from carlactone via the carotenoid pathway, play a crucial role in biological processes [70, 71]. Strigol, the first naturally occurring SL to be discovered, was extracted from cotton root exudates in 1966 [72]. 5-DS is regarded as a precursor for naturally occurring SLs [73]. Previous studies have demonstrated that SLs reduce auxin biosynthesis, lowering local IAA levels and attenuating shoot gravitropism. Thus, mutations in SL biosynthesis or signaling genes suppress the spreading growth phenotype of *lazy1* mutants [74]. In this study, strigol and 5-DS levels were significantly higher in loose B003 compared with compact B010, suggesting that the differential accumulation of SLs (strigol and 5-DS) between loose and compact accessions contributes to their distinct growth phenotypes, potentially through modulation of auxin biosynthesis and shoot gravitropism, as supported by previous studies on SLs [74] and the IAA levels in B003 and B010 in this study.

Calcium serves as a universal secondary messenger that plays a pivotal role in coordinating plant responses to developmental signals and environmental stimuli [75, 76]. In addition, it is essential for maintaining the stability of the cell wall and membranes and modulating physiological processes, including phytohormone signaling pathways [39, 77, 78]. In this study, DEGs were significantly enriched in calcium-mediated signaling, suggesting that this biological process influences FBA in pepper plants through phytohormone signaling pathways and by affecting the structure of cell walls and membranes.

## Conclusions

This study provides the first comprehensive integration of genetic (via GWAS), transcriptomic, hormonal, and cytological analyses to dissect the mechanisms regulating FBA in pepper, leading to a proposed cohesive regulatory framework, in which the underlying gene within the genetic loci modulates key phytohormone pathways, accompanying transcriptomic changes, hormone differences, and cellular alterations that collectively determine branch angle. These findings highlight the crucial role of phytohormones in FBA regulation in pepper and provides valuable information for future breeding strategies aiming to develop pepper varieties with optimal plant architectures.

## Supplementary Information

Supplementary Material 1: Table S1. Materials of pepper (*Capsicum annuum*) used in this study.

Supplementary Material 2: Table S2. Primers used in qRT-PCR.

Supplementary Material 3: Figure S1. Principal component analysis (PCA) of the pepper accessions B010 and B003 based on the gene expression matrix, with three replicates for each accession.

## Data Availability

The RNA-seq datasets generated in this study have been deposited in the NCBI Gene Expression Omnibus (GEO) database under the accession series number GSE289948, which includes sample records GSM8802035 to GSM8802040.

## Abbreviations

28-HBL: 28-homobrassinolide
5-DS: 5-deoxystrigol
ABA: Abscisic acid
BL: Brassinolide
BLUEs: Best linear unbiased estimators
BRs: Brassinosteroids
CDPK24: Calcium-dependent protein kinase 24
CKs: Cytokinins
COG: Clusters of Orthologous Groups
CS: Castasterone
CV: Coefficients of variation
DEGs: Differentially expressed genes
FAA: Formalin/acetic acid/alcohol
FBA: First branch angle
FDR: false discovery rate
FPKM: Fragments per kilobase of exon per million mapped reads
GA1: Gibberellin A1
GA3: Gibberellin A3
GA4: Gibberellin A4
GAs: Gibberellins
GLM: Generalized linear model
GO: Gene Ontology
GWAS: Genome-wide association study
h2: Narrow-sense heritability
H²: Broad-sense heritability
HPLC: High-performance liquid chromatography
IAA: Indole-3-acetic acid
IAA-ASP: Indole-3-acetic acid asparagine
IAM: Indoleacetamide
IAN: Indoleacetonitrile
Indels: Insertion–deletions
iP: isopentenyl adenine
KEGG: Kyoto Encyclopedia of Genes and Genomes
KOG: Eukaryotic Orthologous Groups
LOD: Logarithm of the odds
MLM: Mixed linear model
NR: Non-Redundant
PAS: Periodic acid-schiff
PCA: Principal component analysis
QQ: Quantile–quantile
qRT-PCR: Quantitative reverse transcription PCR
QTL: Quantitative trait locus
RNA-seq: RNA-sequencing
SLs: Strigolactones
SNPs: Single nucleotide polymorphisms
SU: Sympodial unit
SYMs: Sympodial meristems
tZ: trans-zeatin
ZT: Zeatin
