# Supplementary material for: Genetic, hormonal, and transcriptomic analyses highlight the crucial role of phytohormones in first branch angle regulation in pepper
Source: BMC Plant Biol. 2025 Nov 3;25:1492. doi: 10.1186/s12870-025-07499-2 (PMC12581409; doi:10.1186/s12870-025-07499-2)
Supplement: Supplementary file 3 — Supplementary Material 3: Figure S1. Principal component analysis (PCA) of the pepper accessions B010 and B003 based on the gene expression matrix, with three replicates for each accession. [file 12870_2025_7499_MOESM3_ESM.docx]

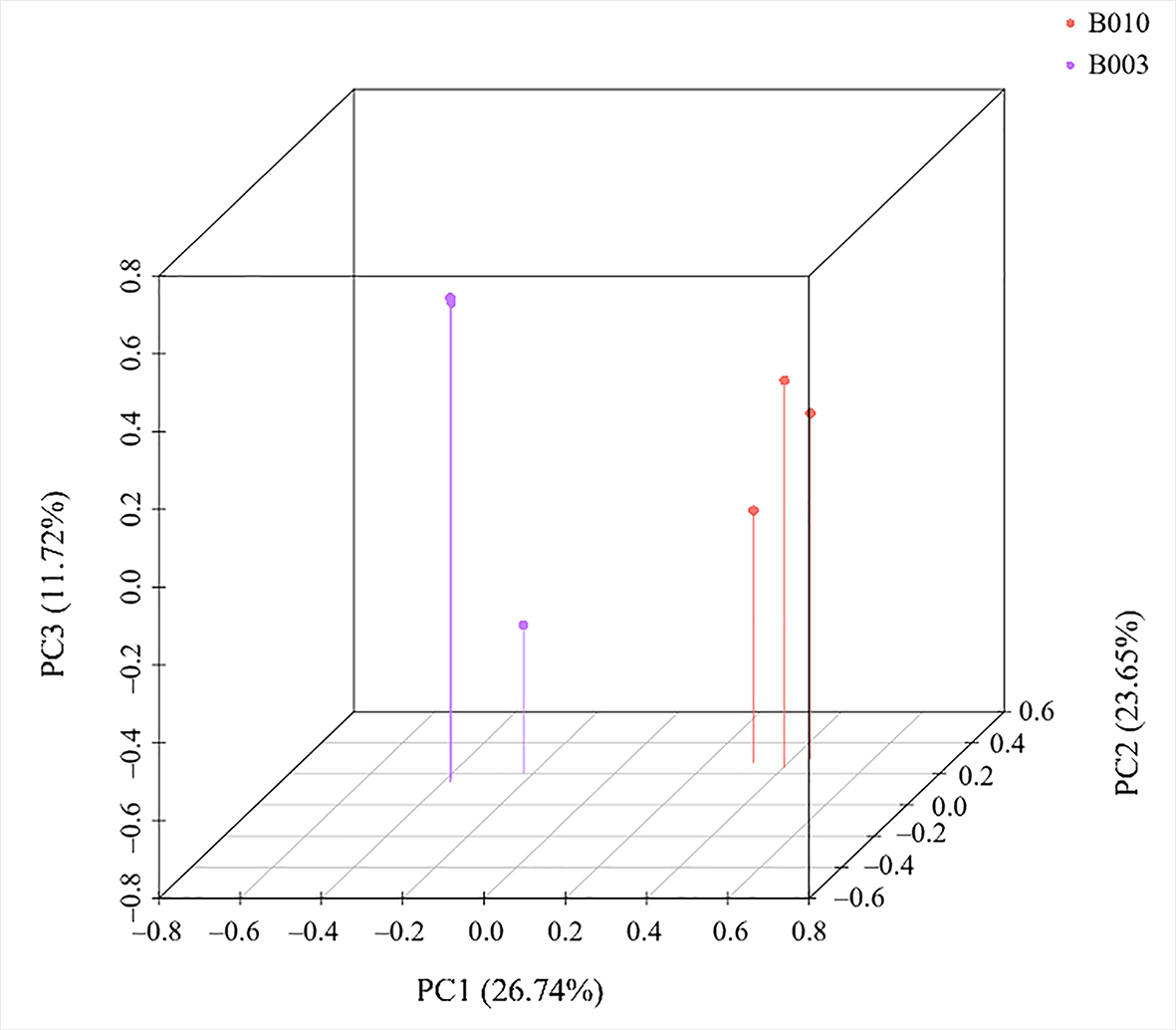


**Figure S1** Principal component analysis (PCA) of the pepper accessions B010 and B003 based on the gene expression matrix, with three replicates for each accession.
